# Perceptions and observations of shared decision making during pediatric otolaryngology surgical consultations

**DOI:** 10.1186/s40463-019-0351-x

**Published:** 2019-06-17

**Authors:** Yolanda Evong, Jill Chorney, Gilanders Ungar, Paul Hong

**Affiliations:** 1 Division of Otolaryngology-Head and Neck Surgery, Department of Surgery, Dalhousie University, Halifax, Nova Scotia Canada; 2 IWK Health Centre, 5850/5920 University Avenue, PO Box 9700, Halifax, Nova Scotia B3K 6R8 Canada

**Keywords:** Shared decision making, Pediatric otolaryngology, Adeontonsillectomy, Tympanostomy tube

## Abstract

**Objective:**

Increased parental involvement in the decision-making process when considering elective surgeries for their children, termed shared decision-making (SDM), may lead to positive outcomes. The objective of this study was to describe perceived and observed levels of SDM during pediatric otolaryngology consultations.

**Methods:**

One hundred and seventeen parents and their children undergoing elective surgical consultations were prospectively enrolled. The visits were videotaped and coded using the Observing Patient Involvement (OPTION) scale. Following the encounter, all participants completed a questionnaire that measured perceived levels of SDM (SDM-Q-9). Surgeons also completed a similar questionnaire (SDM-Q-Doc). Spearman’s correlation coefficient was determined to measure the associations between observed and perceived levels of SDM.

**Results:**

The overall OPTION scores were low (median score of 14 out of 48) and not significantly correlated with perceived levels of SDM (SDM-Q-9, *p* = 0.415; SDM-Q-Doc, *p* = 0.236), surgery type (*p* = 0.197), or patient demographic factors. The OPTION scores were positively correlated with consultation length (*p* < 0.001). There was great variability in the level to which each OPTION items were observed during the consultation (not present in any visits to present in 96.6% of the visits).

**Conclusions:**

Observed levels of SDM were consistently low, but higher levels were observed when the surgeon spent more time during the consultation. Observed levels of SDM did not match perceived levels of SDM, which were consistently rated higher by both caregivers and surgeons.

## Introduction

Many medical conditions can have multiple treatment options. Clear recommendations can make these choices relatively simple, but often the evidence is insufficient to identify a superior treatment course [1]. In pediatrics, parents are often responsible for making these decisions, and uncertainty around decision making has been linked to negative consequences such as decisional conflict, decisional regret, emotional distress, delays in decision making, and post-operative complications [2–8].

Taking a shared approach to decision making has been endorsed as a strategy to improve the informed consent process and health outcomes [2, 3, 9–11]. It has also been suggested that these benefits could potentially influence healthcare systems in a positive manner through reducing the total healthcare costs [13]. *Shared decision making* is a collaborative process in which providers and patients (and family members) work as a team to make joint decisions when more than one reasonable option exists [14]. When practiced, these decisions are evidence based, consistent with best practice guidelines, and tailored to fit the values and circumstances of the individual patient [12]. Previous research has shown low levels of shared decision making across multiple medical specialties [10], but little is known about the extent to which providers facilitate this type of patient/family involvement in pediatric otolaryngology.

The objectives of this study were: 1) to describe the levels of shared decision making that is occurring during pediatric otolaryngology consultations; 2) to compare the perceived and observed levels of shared decision making; and 3) to identify factors which could influence levels of shared decision making.

## Methods

This research was part of a larger mixed-methods study assessing surgical consultations in pediatric otolaryngology [11]. The current results are based on descriptive coding of the video-recordings of surgical consultations using the OPTION scale (see below for details). Previous research conducted by our group used a different coding system (Roter Interaction Analysis System) to quantify interactions between providers and patients/families during medical encounters [15].

### Participants

One hundred and fifty parents of pediatric patients presenting to an academic pediatric otolaryngology clinic were consecutively approached. Twelve parents refused to participate due to time constraints and seven did not wish to be video-recorded. Therefore, 131 total parents were enrolled.

All parents held legal guardianship of the patients and were fluent in English. All patients were less than 6 years of age at the time of consultation and were potential candidates for one of these elective surgical procedures: tympanostomy tube insertion, adenoidectomy, tonsillectomy, and/or adenotonsillectomy.

Three Canadian board certified and fellowship trained pediatric otolaryngologists participated. The surgeons were not fully aware of the purpose of the study and were not trained in delivering shared decision making.

### Procedure

Local Research Ethics Board approval was obtained (IWK Health Centre).

Potential participants were approached by pediatric otolaryngology clinic nurses prior to their consultation with the surgeon. If interested, a research assistant provided details about the study and obtained consent. Participating parents completed a demographic form prior to their appointment and a shared decision making questionnaire (SDM-Q-9) following their appointment. Surgeons completed the physician version of the shared decision making questionnaire (SDM-Q-Doc).

Consultations were filmed using a dual tabled mounted video camera system. Video-recordings were subsequently viewed and coded by two independent raters using the Observing Patient Involvement (OPTION) scale. The primary rater (YE, medical student) coded all recorded encounters, while a secondary rater (GU, graduate student) coded 20% of encounters selected at random to ensure acceptable levels of inter-rater reliability.

### Measures

#### Shared decision making questionnaire-patient version (SDM-Q-9)

This validated 9-item questionnaire was used to measure the parents’ perceived level of shared decision making during the surgical consultation. The total score can range from 0 (no shared decision making) to 100 (extremely high level of shared decision making). The SDM-Q-9 has been used reliably in various medical settings [16].

#### Shared decision making questionnaire-physician version (SDM-Q-doc)

This validated 9-item questionnaire was used to measure the surgeons’ perceived level of shared decision making during the visit. Scoring is the same as SDM-Q-9. The SDM-Q-Doc has demonstrated good internal reliability [17].

#### Observing patient involvement (OPTION) scale

This validated 12-item scale objectively measures the level of shared decision making in medical encounters (primary outcome measure in this study) [18]. Raters assign each scale item a code ranging from 0 (corresponding behaviour was not observed) to 4 (corresponding behaviour was observed at a high standard). The 12 individual scores are then combined to yield a total score between 0 (no observed shared decision making) and 48 (extremely high level of observed shared decision making) per interaction. The OPTION scale has demonstrated high levels of internal consistency, and intra- and inter-rater reliability [18].

### Data analysis

All data was analyzed using SPSS version 24 (IBM Corp.). Interclass correlation coefficient (ICC) was calculated for OPTION scores to confirm acceptable levels of inter-rater reliability (ICC ≥ 0.75) [19]. The OPTION, SDM-Q-9 and SDM-Q-Doc data were not normally distributed; therefore, non-parametric tests were used to assess for significant interactions. Mann-Whitney U testing was used to assess differences in OPTION scores by child gender and parent relation to child. Kruskal-Wallis testing was used to assess OPTION scores by child and parent age, parent ethnicity, parent marital status, parent education level, and household income. Spearman’s ρ correlations were used to examine the relationship between OPTION scores and perioperative variables.

## Results

### Participants

Of the 131 parents enrolled, 117 were included in the analysis (2 withdrew, 7 had incomplete data, and 5 were excluded because surgery was not an option for their child). Participants’ baseline information is provided in Table 1.

**Table 1:** Participants’ baseline information

	Parent	Child
Age (mean years for parents; mean months for children)	Female: 33.16 (range 19–44, SD 5.0)	35.02 (range 8–72, SD 15.5)
	Male: 35.33 (range 19–53, SD 6.1)	
Sex	Female: 92	Girls: 43
	Male: 25	Boys: 74
Marital Status	Married or common-law: 104	
	Not married: 22	
Ethnicity	Caucasian: 105	
	African Canadian: 6	
	Other: 6	
Parent Education	High school or less: 19	
	College/undergraduate: 70	
	Graduate degree or higher: 27	
Household Income (Canadian dollars)	Less than $20,000: 6	
	$20,000–$50,000: 21	
	$51,000–$100,000: 54	
	More than $100,000: 38	

*SD*-standard deviation

Three fellowship trained pediatric otolaryngologists, 2 males and 1 female, participated in the study (mean of 10.74 years in practice; range 5–15 years).

### Levels of shared decision making

#### Perceptions of shared decision making

The total median SDM-Q-9 score was 85.12 [range 29.36–100, interquartile range (IQR) 17.59] and the total median SDM-Q-Doc score was 79.63 (range 44.44–100, IQR 12.96). The SDM-Q-9 and SDM-Q-Doc were significantly positively correlated (Spearman’s ρ = 0.242, *p* < 0.009).

#### Observations of shared decision making

The ICC was 0.92, indicating excellent reliability between the primary and secondary OPTION coders [19].

The total median OPTION score was 14 out of 48 (range 0–30, IQR 12) per encounter. The observed frequencies of individual items on the OPTION scale are reported in Table 2. “The clinician explains the pros and cons of the options to the patient” (96.6%) and “The clinician checks that the patient has understood the information” (95.7%) were observed the most often. “The clinician assesses the patient’s preferred approach to receiving information to assist decision making” (0%) and “The clinician elicits the patient’s preferred level of involvement in decision making” (48.7%) were observed considerably less frequently. Median consultation scores of individual OPTION items are also reported in Table 2. These varied from 0 (“The clinician assesses the patient’s preferred approach to receiving information to assist decision making”) to 3 (“The clinician explains the pros and cons of the options to the patient”) out of 4.

**Table 2:** Individual OPTION items by frequency, percentages and median consultation score

OPTION item for parents	Number of consultations in which the competence is demonstrated	Percentage of visits that include the competence	Median score per consultation
The clinician draws attention to an identified problem as one that requires a decision-making process	106	90.6	2
The clinician states that there is more than one way to deal with an identified problem	106	90.6	1
The clinician assesses the patient’s preferred approach to receiving information to assist decision making	0	0	0
The clinician lists “options”, which can include the choice of “no action”	95	81.2	2
The clinician explains the pros and cons of the options to the patient	113	96.6	3
The clinician explores the patient’s expectations (or ideas) about how problems are to be managed	83	70.9	1
The clinician explores the patient’s concerns (fears) about how the problems are to be managed	106	90.6	1
The clinician checks that the patient has understood the information	112	95.7	1
The clinician offers the patient explicit opportunities to ask questions during the decision-making process	108	92.3	1
The clinician elicits the patient’s preferred level of involvement in decision making	57	48.7	0
The clinician indicates the need for a decision-making (or deferring) stage	102	87.2	1
The clinician indicates the need to review the decision (or deferment)	79	67.5	2

There was no significant difference in total OPTION scores by any of the baseline demographic variables and most perioperative characteristics, with the exception of a positive correlation between total OPTION scores and consultation length (Table 3). There was no significant correlation between OPTION scores and SDM-Q-9 or SDM-Q-Doc scores.

**Table 3:** Associations between baseline characteristics and OPTION scores

Parent Factors	Spearman’s rho^†^ or Mann-Whitney U and Z scoreª or Kruskal-Wallis (Chi-square, df)^¥^	Significance
Parent age	0.05^**†**^	0.793
Parent gender	3.056, 3^¥^	0.764
Ethnicity	4.345, 5^¥^	0.818
Parent education	6.496, 5^¥^	0.261
Marital status	1.250, 4^¥^	0.945
Family income	1.015, 5^¥^	0.363
Consulting surgeon	1.154, 5^¥^	0.450
Time with surgeon	0.561^**†**^	< 0.001
Type of surgery	0.004^**†**^	0.964
Previous surgical experience (yes or no)	645.5, −0.015**ª**	0.211
Parent perceptions of shared decision making (SDM-Q-9)	0.076^**†**^	0.415
Surgeon perceptions of shared decision making (SDM-Q-Doc)	0.110^**†**^	0.236

*df*-degrees of freedom

†Spearman’s rho

ªMann-Whitney U and Z score

^¥^Kruskal-Wallis (Chi-square, df)

## Discussion

Elective surgical procedures are frequent in pediatric otolaryngology and more than one reasonable treatment option can exist for common conditions. For instance, some children with sleep disordered breathing can improve over time without surgical intervention [20]. Using a collaborative approach with parents and providers to make these treatment decisions, termed shared decision making, has been linked to many positive outcomes [2, 3, 9, 11]. However, previous research has demonstrated low levels of shared decision making across multiple medical specialties [10]. Given the high prevalence of elective surgeries in pediatric otolaryngology and the benefits associated with shared decision making, more research is needed to improve our awareness and understanding. Furthermore, there is a need for research focused on the barriers to shared decision making in otolaryngology and the possible methods by which to better implement shared decision making in routine clinical practice [21].

Shared decision making describes a collaborative approach in which patients/family members and healthcare providers work together to make a medical decision based on up-to-date evidence while considering patient values and preferences [13]. Previous studies with pediatric patients show that parents may not be as involved in decision making as they would like, and there appears to be differences in how parents and physicians perceive shared decision making. Specifically, parents tend to view shared decision making as an equal partnership, whereas providers reported that they viewed shared decision making as a way to convince parents to follow recommended management plan [22].

Overall, this study found consistently low levels of shared decision making in pediatric otolaryngology consultations. Similar results were previously found in a study conducted by our group with older pediatric patients and their caregivers [23]. All together, these findings suggests that providers are not adequately involving parents in the decision making process. This is particularly important in terms of barriers to shared decision making as the literature shows that many providers believe that they are already practicing shared decision making, and thus there is no need for change [22, 24, 25]. Therefore, providers must first acknowledge that there is minimal shared decision making in routine clinical practice before actions can be taken to address this deficiency.

Although the overall shared decision making levels were low, healthcare providers were more likely to exhibit some collaborative behaviours over others. Specifically, surgeons explained the pros and cons of the treatment options to parents and confirmed understanding during most consultation visits, which are two behaviours that are focused on *providing* information. Conversely, surgeons rarely elicited the parents’ preferred method for receiving information or their preferred level of involvement in the decision making process, which are two highly patient-centred behaviours used to *gather* information. These findings are consistent with a recent systematic review which found that the most patient-centred OPTION behaviours (i.e., those involving eliciting patient preferences and subsequently tailoring the provider’s counselling style to fit these needs) were performed the least frequently across 33 studies [10]. Moreover, one study found that provider utterances related to giving information were twice as frequent as those related to gathering information [26]. These findings imply a disproportionately unidirectional exchange of information from clinician to patient and highlight a need for increased patient involvement during consultations. Future research and interventions should target these behaviours, as progress in this area has the capacity to markedly increase levels of shared decision making and improve patient centered care.

The perceived levels of shared decision making were higher than the observed levels. This finding was consistent with prior research performed by our group [22] and in other medical settings [5, 27, 28]. The implication could be that current shared decision making instruments may not be capturing the same construct or that a ceiling effect may be present when measuring *perceptions* of shared decision making. Moreover, with these inconsistencies between the parent, provider and observer perspectives, it is important to determine which viewpoint is most strongly related to outcomes. It is well documented that parents who feel more involved in the decision-making process experience less decisional conflict and report higher levels of satisfaction [4–6, 11], but the relationship is complex and the contributions of actual behaviour has not been examined as thoroughly. Clearly, more research is needed to determine the relative influence of perceptions and actual behaviour on both parent and patient outcomes.

The observed levels of shared decision making increased with consultation length. That is, the total OPTION scores were generally higher when surgeons spent more time interacting with parents. Previous research has noted similar findings [29–33] and further supports a recent systematic review, which concluded that longer consultations usually coincide with higher levels of shared decision making in various medical specialties [10]. In fact, this review article identified consultation length as one of the top two variables most consistently correlated with higher levels of shared decision making. However, it is important to note that in our study, while shared decision making did *relatively* increase with consultation length, the *actual* levels remained low. This suggests that longer consultation time alone may not be sufficient to achieve satisfactory levels of shared decision making in clinical practice, and further research is needed to determine the potential contributions of other variables to increase the overall level of shared decision making in pediatric otolaryngology.

The level of observed shared decision making was independent of all other variables including previous surgical experience, type of surgery, and consulting surgeon. Furthermore, shared decision making levels did not differ with respect to parental education, ethnicity, marital status, age, or household income. Interestingly, previous studies have shown mixed results on this matter. Some studies corroborate our findings [30, 32], while others demonstrated a correlation between shared decision making and some demographic factors, such as education and ethnicity [33–35]. The explanation for this discrepancy remains unknown and more research is needed to determine the true manner of interaction, or lack thereof, between shared decision making and these variables [2, 29, 36–38].

There are several limitations worth noting. First, this study was carried out at one centre with a total of three providers. Different geographical areas and providers may interact uniquely, which can reduce the generalizability of the study findings to other populations. However, the current results are still in keeping with similar studies done in other areas of medicine [10]. Second, both parents and providers were aware that the consultation was being recorded. Although both parties were unaware of the purpose of the recordings (i.e., to assess the level of shared decision making), the simple knowledge of being observed may have caused them to modify their behaviours in some way.

## Conclusion

Observed levels of shared decision making were low during pediatric otolaryngology consultations in this study. However, there was variability in the level to which each individual OPTION item was present, indicating that certain collaborative behaviours have more room for improvement. Providers should be aware that patient-centred behaviours such as assessing their desired levels of involvement are often lacking in elective surgery consultations, and that perceptions of adequate shared decision making are not always associated with actual observed behaviours. Shared decision making has garnered a lot of attention, but the uptake has been poor and implementation into routine clinical practice remains unrealized. Efforts should be made to increase levels of shared decision making in pediatric otolaryngology moving forward.

## Data Availability

The dataset generated during and/or analyzed during the current study are available from the corresponding author on reasonable request.
